# Whole-body vibration exposure and chronic low back pain among heavy machine operators and long-haul truck drivers in Tanzania: a cross-sectional study

**DOI:** 10.3389/fpubh.2026.1735367

**Published:** 2026-03-16

**Authors:** Israel Nyarubeli, Gloria Sakwari, Suleiman Chombo, Susan Reuben, Naanjela Msangi, Robert Duguza, Bryan Francis, Simon Lwaho, Abdulsalaam Omar, John Mduma

**Affiliations:** 1 Department of Psychosocial Sciences, University of Bergen, Bergen, Norway; 2 School of Public Health and Social Sciences, Muhimbili University of Health and Allied Sciences, Dar es Salaam, Tanzania; 3 Workers Compensation Fund (WCF), Dar es Salaam, Tanzania; 4 Occupational Safety and Health Authority, Dar es Salaam, Tanzania

**Keywords:** low back pain, mining, occupational exposure, Tanzania, transportation, truck drivers, whole-body vibration

## Abstract

**Background:**

The Workers Compensation Fund (WCF) in Tanzania has noted an increased rate of injury claims for work-related musculoskeletal disorders (WRMSDs), particularly chronic low back pain among truck drivers and heavy machine operators. These trends indicate a likelihood of occupational exposure to whole-body vibration (WBV), nevertheless local and contextual evidence is needed to guide policy and decision-making. This study aimed to quantity and characterize the WBV exposure and examined its association with self-reported 12-month chronic low back pain among heavy machine operators and long-haul truck drivers in Tanzania.

**Methods:**

We conducted an analytical cross-sectional study of 632 participants in 2023 with a total of 141 WBV measurements. The WBV was measured at the seat-operator interface and analyzed using the International Organization for Standardization - ISO 2631-1:1997 to derive 8-h equivalent daily exposure, A(8). A standardized and pretested questionnaire was used to collect information on WRMSDs related factors. Data were analyzed using regression models and Chronic low back pain was defined as self-reported low back pain during the preceding 12 months.

**Results:**

The mean A(8) was 0.63 m/s^2^ (SD = 0.20) in the mining and 0.51 m/s^2^ (SD = 0.08) in the transportation. In the mining sector, 71% (*n* = 41) of measurements were ≥0.5 m/s^2^ A8 - exposure action value (EAV). The 12-month prevalence of chronic low back pain was 42.5%. In adjusted models, WBV exposure ≥0.5 m/s^2^ was associated with higher prevalence of chronic low back pain (adjusted RR 1.22; 95% CI 1.01–1.47), as were working in the mining sector (adjusted RR 1.36; 95% CI 1.05–1.75), increase in body mass index (BMI) (adjusted RR 1.02; 95% CI 1.00–1.04) and reporting leg pain symptoms (adjusted RR 2.36; 95% CI 2.04–2.73).

**Conclusion:**

A significant number of measurements exceeded the Directive 2002/44/EC EAV, and a substantial proportion of workers reported chronic low back pain, suggesting a potential relationship. Preventive interventions to reduce WBV exposure could benefit heavy machine operators and truck drivers. Longitudinal studies with repeated measurements are warranted to better characterize exposure variability and WRMSDs.

## 1 Introduction

Whole-body vibration (WBV) is a significant occupational hazard, and exposure is prevalent in the mining and long-distance transportation sectors in both developed and developing countries (1). WBV exposure often arises from elevated vibrations and/or prolonged operation of vibrating heavy machinery and long-haul trucks, leading to adverse health outcomes such as work-related musculoskeletal disorders (WRMSDs) affecting the back, cervical region, and upper extremities, with potential impacts on physical performance in job tasks (2). The most frequently reported conditions associated with WBV exposure include chronic low back pain, which may manifest as persistent pain, discomfort, and reduced mobility among affected workers (3–5). These disorders can adversely affect quality of life and contribute to absenteeism, reduced productivity, and increased healthcare costs, while the psychological burden of chronic pain may further compound health outcomes (6–8). Globally, musculoskeletal conditions account for approximately 17% of years lived with disability, and back pain is a major contributor to disability including in precarious work settings where prevalence has been reported as high (9–12). In developing countries, including Tanzania, WBV-related health effects may be amplified by long working hours, rough terrain and road conditions, variable ergonomic factors, limitations in exposure control and surveillance factors that align with the increasing Workers Compensation Fund (WCF) claims for chronic low back pain among truck drivers and heavy machine operators.

Studies suggest that operators and drivers in the mining and transportation sectors are frequently subjected to high levels of WBV at work (13–15). For instance, in the transportation sector, long-haul truck drivers in North America have been reported to experience daily vector sum for WBV exposure, A(8) levels exceeding the Health Guidance Caution Zone (HGCZ) established by standards ISO 2631-1:1997 and ISO 2631-5 while working in an extended working hour schedule averaging 13-h per day and 80 h a week (16). This prolonged exposure level and duration was related to increased symptoms of work-related ill health, particularly WRMSDs of low back, placing this occupation among the high-risk categories for nonfatal injuries (16). These findings underscore the need for monitoring and preventing WBV exposure among truck drivers, where the combination of individual factors (age, anthropometry, sex), work conditions (long work hours across varying road conditions, different truck models and sizes, including suspensions), inadequate ergonomic practices, and psychosocial work hazards may aggravate health risks (10, 15). Similar observations have also been documented among heavy mobile equipment operators in the mining sector. The common job groups for heavy machine operators include graders, dump trucks, dozers, excavators and Loader operators. A study conducted in India reported that more than 90% of mining heavy machinery operators were exposed to WBV levels exceeding the HGCZ established by the ISO 2631-1:1997 (17). Nevertheless, studies are limited to informing employers, workers, and policy makers as well as planning and implementing effective interventions in workplaces. More often than not, the technical equipment for conducting WBV surveillance at workplaces is not available.

The Tanzania WCF operates within International Labor Convention No. 102 of 1952 on the Minimum Standard for Social Security, which forms the framework for provisions of benefits for work-related injuries, diseases, and even deaths in which several benefits may be covered (18). Furthermore, benefits are payable to eligible employees who sustain injury because of occupational accidents or disease. In the extreme event that an occupational accident or disease results in the death of an employee, a benefit is paid to his dependents. In practice, when an employee sustains injuries because of an occupational accident or disease, the employee may be entitled to medical aid, rehabilitation services, temporary and permanent disability benefits, constant attendant care grants, and the death benefits (19). However, the guideline for the diagnosis of occupational diseases requires that claims submitted for compensation be objectively assessed, i.e., there should be, among other things, evidence of epidemiological literature and workplace exposure information that provides empirical evidence suggesting a positive association between exposure to such factors (WBV in this case) and the occurrence of that disease or condition, such as chronic low back pain (20).

Despite the existence of international standards and regulatory frameworks aimed at mitigating occupational WBV exposure, implementation and compliance remain inconsistent across countries and industries. ISO 2631-1: 1997 provides general exposure guidance using frequency-weighted acceleration metrics, whereas ISO 2631-5:2018 offers a complementary approach intended for exposures containing repeated mechanical shocks with outputs related to lumbar spine (vertebral end-plate) compressive loading in seated operators (21–24). In addition, regulatory frameworks such as the European Physical Agents (Vibration) Directive (2002/44/EC) specify enforceable exposure “action” and “limit” values [e.g., EAV 0.5 m/s^2^ A(8) and ELV 1.15 m/s^2^ A(8)] to guide prevention and control (25). In developing-country contexts, limited training and occupational health resources may further constrain effective implementation, and the increasing claims for low back and other musculoskeletal disorders reported to the WCF in Tanzania underscore the need for locally grounded WBV exposure evidence to inform policy and prevention (26). Therefore, the study aimed to quantify and characterize the WBV exposure and examined its association with self-reported 12-month chronic low back pain among heavy machine operators and long-haul truck drivers in Tanzania was conducted.

## 2 Materials and methods

### 2.1 Study design

This was an analytical cross-sectional study involving occupational exposure measurements of the WBV among truck drivers and heavy machine operators in the transportation and mining industries, respectively. The study aimed at characterizing the WBV exposure levels among truck and heavy mobile machine operators using ISO 2631-1:1997 and comparing the established occupational exposure limit values; and determining association between measured WBV exposure and self-reported 12-month chronic low back pain with relevant covariates. The field data collection was conducted between May and July 2023. Reporting follows the STROBE guideline for cross sectional studies (Supplementary material-1) (27).

### 2.2 Study participants

The study included participants who were truck drivers and heavy mobile machine operators in the transportation and mining industries, respectively, in mainland Tanzania. In this study, we identified and included eight (8) companies that were registered for carrying out both national and international cargo transportation that generally represents a typical work situation for this sector, and two (2) large mining operations (mine “A” and “B”), those with permanent employment or long contract employment renewable (at least a year), and those with truck-driver matching, i.e., those trucks that were assigned to the same driver or operator throughout employment except during sick or normal leave or perhaps during emergencies. In the transportation sector, long-haul trucks were selected from participating companies based on operational availability during the measurement period and stable truck–driver assignment (“truck–driver matching”). For each measured truck, we recorded key characteristics relevant to WBV transmission (e.g., axle configuration, seat or suspension condition, and recent maintenance history) using a standardized vehicle checklist. The study included 632 participants and 141 WBV measurements.

#### 2.2.1 Inclusion criteria

We included adult participants (above 18 years of age), truck drivers who were permanently employed or had a contract of at least 1 year, with a driving license (professional drivers or operators).

#### 2.2.2 Exclusion criteria

We excluded truck drivers or heavy mobile machine operators with clinically diagnosed chronic WRMSDs (to reduce potential bias and improve study sample homogeneity) and those on leave during the study period.

### 2.3 Whole-body vibration exposure measurements

Whole-body vibration exposure measurements were conducted according to the ISO 2631-1:1997 standard, where measurements were carried out at the operator–seat interface of the truck with triaxial seat pad accelerometer equipment using a calibrated vibration analyzer (PCE-VM31 and Svantek 106A). The seat pad accelerometer was placed such that the x-axis was in the sagittal plane, the y-axis was in the coronal plane, and the z-axis was in the vertical plane. The equipment used in the WBV measurements, i.e., PCE-VM31 and Svantek 106A, was factory—calibrated and field checked (28).

During WBV measurement, a prepared vehicle checklist was used to gather key information related to vehicle characteristics (model, make), including the cabin (seat condition, suspension) and haul road conditions. The sampling time covered a full operation cycle comprising activities such as drilling, loading and shoveling to the haulage of mined ore materials. Sampling for mining heavy mobile equipment was conducted in Dump Truck (DT) (including its operational subcategories such as articulated dump truck (ADT), Dozer, Loader, Grader, and Boger models).

All WBV measurements were conducted during the day shift for logistical, operational and safety reasons. Measurements were intended to capture a full representative operating cycle under normal production conditions. Because mining tasks and haulage cycles are broadly similar across day and night shifts at the study sites, day-shift measurements were considered representative at the equipment or job-category level.

A larger sample size with full shift measurements can estimate WBV exposures and reduce the risk of random errors or biases (29). Nevertheless, conducting occupational WBV exposure assessments on heavy mobile equipment operators and truck drivers was time-consuming and resource-intensive and required relatively more measurement devices to accomplish the planned study tasks. The ISO 2631-1:1997 does not provide minimum sampling duration, rather it requires that the measurement period be sufficient to achieve reasonable statistical precision (22). The European Commission—non-binding practical guidance for workplace WBV assessment recommends that, where feasible, vibration measurements be conducted for at least 20 min and guide that time to be recorded together with relevant work duration and related characteristics for representativeness (30, 31). The characteristics of our study population included extended work shifts of more than 8 h with breaks in between for most truck drivers. Therefore, an average measurement duration of approximately 30 min was used for this study while ensuring that the recorded period captured a representative operating cycle (31). We performed 41 and 100 measurements from the mining and transportation industries, respectively (Table 1). During sampling, we conducted single measurements for several models (DT-Model 3, Model-IT and Model-CT). This was because they were the only ones available. The mean measurement duration was 31:59 (mm:ss) in mining (range across models: 27:28–42:09) and 34:00 in transportation (range: 27:46–42:19) (Table 1). Measurements were planned to capture a representative operating cycle under typical production conditions; in mining this included both loaded and unloaded phases. Repeated cycles for the same individual driver or operator were not conducted, because exposure was assigned at the vehicle/equipment model (job-category) level. The interviews were conducted using prepared structured questions and observation checklists. The questionnaire included information on shift duration, i.e., the time used for truck driving (excluding breaks), lifting load, driving posture, road condition (terrain), and average speed for each road condition (29). WBV exposure was assigned at the job-category level. A(8) was calculated for each WBV measurement according to ISO 2631-1:1997 and then summarized by equipment or truck model for the transportation and by mine site for the mining sector. Each participant was assigned the corresponding stratum-level exposure estimate based on the vehicle or equipment model they operated according to NIOSH occupational exposure sampling strategy (32).

**Table 1:** Number of whole-body vibration measurements conducted during the study in Tanzania (*N* = 141).

**Mining industry**				**Transportation industry**			
**Machine and truck model**	**Number measurements**	**Measurement duration**		**Truck model**	**Number of WBV measurements**	**Measurement duration**	
		**Average duration (mm:ss)**	**Range (mm:ss)**			**Average duration (mm:ss)**	**Range (mm:ss)**
DT model−1	10	31:39	27:28–34:39	Model- BT	28	32:23	27:59–35:41
DT model- 2	8	32:56	28:53–39:43	Model- VT	16	34:28	30:39–41:30
Excavator	5	31:02	28:11–37:49	Model- HT	30	35:39	29:26–37:59
Loader	3	29:40	27:51–30:53	Model -ST	13	34:45	30:17–42:09
Articulated dump truck	11	31:39	29:26–42:09	Model -TT	3	33:18	30:31–42:19
Dozer	3	29:39	27:46–32:53	Model -FT	5	28:47	27:47–29:53
DT model- 3	1	35:19	–	Model -NT	3	33:33	31:48–35:19
				Model -IT	1	32:07	–
				Model -CT	1	28:53	–
Total	41	31:59	27:28–42:09		100	34:00	27:47–42:19

We administered a structured WBV health-surveillance questionnaire adapted from the EU BIOMED “Guidelines for Whole-Body Vibration Health Surveillance” (33), which provides a standardized protocol and draft instrument for workers exposed to vehicle-related vibration. To assess musculoskeletal symptoms, we incorporated selected items from the Nordic Musculoskeletal Questionnaire (NMQ) with 7-day (acute) and 12-month (chronic) recall periods for the low back, neck, and shoulder regions (34). Items addressing perceived vibration direction, driving/operating hours and breaks, sitting posture, and manual handling demands were aligned with ISO 2631-1:1997 concepts and axis conventions. The questionnaire was pre-tested prior to field deployment and covered: (i) sociodemographic characteristics [age, sex, education level, and body mass index (BMI)]; (ii) occupational history (job title, tenure, typical shift duration, and task characteristics); and (iii) self-reported health history and current lifestyle exposures relevant to vibration exposure, including symptoms in the low back, legs, neck, and shoulders, as well as other reported disorders. The instrument has been used in occupational settings and has demonstrated acceptable measurement properties across multiple contexts (34). We operationally defined chronic low back pain as self-reported low back pain or symptoms during the preceding 12 months (35).

### 2.4 Work schedule in the mining sector

Two large mining sites were involved: mining site “A” and mining site “B”. Both mining sites work for 12 h per shift. The shift patterns for mining site “A” were as follows: open pit mining work schedule was two (2)-day shifts (12 h, one lunch break for 30 min) followed by 2-night shifts (30 min dinner, two breaks at 3:15 am and 5:00 am) and then 4 days off duty. For underground mining, the arrangement was a 4-day shift followed by 4 days off duty and then a 4-night shift. For mining site “B”, the shift pattern was a 7-day shift, followed by a 7-night shift and then a 7-day shift.

### 2.5 Characteristics of heavy mobile machinery and long-haul trucks

In the transportation sector, WBV measurements involved long-haul trucks on paved roads, with drivers having different driving behaviors and postures. The trucks pulled trailers/containers or loose cargo over hundreds to thousands of kilometers to and from Dar es Salaam, Tanzania and the Democratic Republic of the Congo or Republic of Rwanda and some trucks to Zimbabwe. The truck carrying rated capacity was approximately 30 tons on average. The actual loading ranged between 25 and 40 tons. Most of the truck seats had air/pneumatic/hydraulic suspensions and were in good condition, while few of them were defective and rigid. All trucks were wheeled, always pulling trailers behind the cabin, most of which had 6 axles (one in front, tractor unit and three at the trailer or rear). Most trucks reported having a regular schedule of maintenance, mostly after every 20,000 km, and were reported to travel at varying speeds of 10–120 km/hr, depending on the road conditions (36). However, there are some in-between-truck model variations in characteristics, such as suspension systems (37, 38).

In the mining sector, WBV measurements were taken when the mobile equipment machines were both loaded and unloaded. This was done to complete a full task cycle, with mobile equipment moving on unpaved mines hauling roads at varying speeds and time periods. These variations depended on the planned activity of that particular day, the location of mining and the dumping distance. The tasks performed included ore-drilling, shoveling, loading, haul road maintenance and haulage of ore materials.

### 2.6 Work schedule in the transportation sector

The work pattern for the transportation sector varies depending on the company and task arrangements. In general, the driving hours are averaged into 10 h with flexibility and breaks in between starting from approximately 5 a.m., to approximately 6 to 8 p m. During the sampling period, a significant number of drivers reported driving for an average of 450–500 km per day.

### 2.7 Data management and analysis

All data were collected by the researchers of this study and handled with care. Truck drivers' names as well as specific truck model real names were stored in a separate datasheet. Each research participant was assigned an identification code linked to his/her specific information. Paper forms were stored securely and linked only via identification codes.

Participant characteristics were summarized using means with standard deviations for continuous variables and counts with percentages for categorical variables. WBV exposure distributions were summarized overall and by sector and mine site. The proportion of measurements exceeding the exposure action value (EAV) of 0.5 m/s^2^ A(8) was reported.

For WBV exposure, the raw data file was processed according to ISO 2631-1:1997 by computing the weighted average (8-h equivalent) vibration level A(8) for each truck driver to account for differences in exposure time using the vehicle vector sum root mean square (rms) vibration level, A_w_ and estimated exposure time. The corresponding weightings (*x*-axis = W_d_, *y*-axis = W_d_, and *z*-axis = W_k_) and multiplying factors (*x*-axis, *K* = 1.4; *y*-axis, *K* = 1.4; and *z*-axis, *K* = 1) were applied. Analysis of the whole body vibration measurements was done using an excel worksheet delivered with the equipment and available at https://www.products for 'PCE-V M1' | PCE Instruments (pce-instruments.com). An Excel worksheet was used to calculate exposures from the measurements into 8-h equivalents. The results were reported in acceleration values (m/s^2^) and then compared with the standards for the WBV occupational exposure action value as established by Directive 2002/44/EC. Contextually, some regulatory bodies and authorities such as the Tanzania Bureau of Standard (TBS) 2018 Mechanical Vibration and shock—Evaluation of human exposure to whole-body vibration TZS 1398-1:2018 – ISO 2631-1:1997 have adopted the use of this international standard.

Factors of WBV exposure were examined using multivariable linear regression models for the mining and transportation sectors separately. In this analysis, the WBV exposure [A(8)] were entered as the dependent variable and measured as a continuous variable. The variable was transformed to the natural logarithm of vibration exposure—in [A(8)] to ensure that the normality assumption was met. The independent variables included the type of truck model, tire type (wheel/chain) and rated load (in tons). The models in the mining sector included DT model-1, DT model-2, DT model-3, ADT, excavator, loader and Dozer. In the transportation sector, the included models were model VT, model BT, model HT, model ST, model FT, model TT, model CT, model NT, and model IT. Multivariable regression models separated by the employment sector (mining and transportation sector) were constructed. Variables had differing sample sizes due to item non-response (missing data). For regression analyses, we used complete-case analysis for each model. The analytic entries for each model were reported in the corresponding tables.

Initially, Univariable analyses were performed for several factors/covariates, including sociodemographic factors (age, duration of work, BMI) and work organization factors (driving distance, resting hours), to determine potentially significant variables. Any variable that had a *p* ≤ 0.2 was included in the multivariable model. Variables related to truck models, despite having *p* ≤ 0.2, were also included in multivariable models for the purpose of analysis. In these Univariable analyses, no demographic or work organizational factors that showed significant results were left out. In addition, there was a high correlation between the age of the worker and the years of employment. In the final analysis, the variable “years of employment” was retained because it had relatively greater significance.

Two multivariable models were produced from splitting analysis, one for the mining sector and the other for the transportation sector. The essence of the analysis assumed that the types of models used in the mining sector are different from those used in the transportation sector. In the multivariable analysis, variables for which the *p*-value < 0.05 were significantly related to the natural logarithm of whole-body vibration exposure.

To examine associations between WBV exposure and self-reported low back pain, we fitted modified Poisson regression models with robust variance estimation for binary outcomes, providing adjusted relative risks (RR) with 95% confidence intervals in the transportation and mining sectors. In these analyses, the dependent (outcome) variable was chronic low back pain measured as a binary variable (0 = no low back pain, 1 = yes, with low back pain). Independent The primary exposure i.e., the WBV was categorized using the EAV threshold [ < 0.5 vs. ≥0.5 m/s^2^ A(8)]. The main model was adjusted for *a priori* covariates and potential confounders including number of resting breaks as a categorical variable (1 = “0 to two times”; 2 = “three times or more”), acute leg pain symptoms (0 = “no”; 1 = “yes”), daily activity that included the use of the backbone, neck and shoulder as a binary variable (0 = “no”; 1 = “yes”), and age and body mass index, which were expressed as continuous variables. Additional variables for the chronic back pain model included chronic leg pain symptoms as a binary variable (0 = “no”, 1 = “yes”), physical exercise (0 = “no”, 1 = “yes”), and the employment sector (1 = “mining sector” 2 = “transportation sector”). Statistical significance was defined as *p* < 0.05 (two-sided). All analyses were conducted using STATA version 17 (StataCorp, College Station, TX, USA).

### 2.8 Ethical clearance

The ethical approval for this study was obtained from the Muhimbili University of Health and Allied Sciences Institutional Ethical Review Board (MUHAS-REC-09-2023-1894). Permits to collect primary data from research participants, including truck photos, were requested from their respective transportation and mining companies. The study participants provided informed consent, and the information collected was treated with confidentiality.

## 3 Results

### 3.1 Demographic characteristics

This study included 632 participants, 75% of whom were from the transportation sector. The participants had a mean age of 42 (SD = 9; range 22–71) years, and 99% were male. The average number of years employed was 6 (SD = 5.7). Approximately 59% reported doing physical exercise, and the majority (approximately 73%) reported having ≤ 2 (periods of) resting breaks of approximately 30 min while driving, especially truck drivers with long-haul routes. Approximately 34% of the drivers and heavy machine operators had a normal weight, 42% overweight, and 23% obese (Table 2).

**Table 2:** Demographic characteristics of truck drivers and heavy machine operators in the transportation and mining sectors in Tanzania (*N* = 632).

**Variable**	**Count**	**Percent**
**Sex (*****n*** = **632)**		
Male	624	98.7
Female	8	1.3
**Educational level (*****n*** = **632)**		
Primary level	360	57.0
Secondary level	211	33.4
College/vocational training	61	9.7
**Employment sector (*****n*** = **632)**		
Mining	158	25.0
Transportation	474	75.0
**Do physical exercises (*****n*** = **632)**		
Yes	369	58.5
No	259	40.9
Missing	4	0.6
**Backbone and shoulder activities (*****n*** = **632)**		
Yes	69	11.0
No	543	85.9
Missing	20	3.1
**Number of resting breaks (*****n*** = **632)**		
≤ 2 times	460	72.8
3 times or more	172	27.2
**Chronic leg pain (*****n*** = **632)**		
No	587	92.9
Yes	41	6.5
Missing	4	0.6
**Age group (*****n*** = **632)**		
22–39	267	42.0
40–71	365	58.0
**Body mass index (BMI) (*****n*** = **632)**		
< 18.5	5	0.79
18.5 to < 25	214	33.86
25 to < 30	265	41.93
>30	148	23.42
Body mass index: mean (SD) (*n* = 632)	27.0 (4.8)	
Years employed: median (IQR) (*n* = 632)	1 (1, 3)	
**Years employed (*****n*** = **629)**		
0–5 years	393	62.5
6–40 years	236	37.5

### 3.2 Whole-body vibration exposure levels among truck drivers and heavy machine operators in the transportation and mining sectors

A total of 141 WBV measurements were conducted in the two sectors, i.e., 41 from the mining sector (22 mining site A, 19 mining site B) and 100 from the transportation sector (Table 1). Overall, mean 8-h equivalent WBV exposure in mining was 0.63 m/s^2^ A(8) (SD = 0.20), with site specific means of 0.68 (SD = 0.21) m/s^2^ at mine A and 0.71 (SD = 0.24) m/s^2^ at mine B. The mean crest factor in mining was 7.12 (SD = 2.21). In transportation sector, mean WBV exposure was 0.51 (SD=0.08) m/s^2^, with a mean crest factor of 4.93 (SD = 1.88) (Table 3). A total of 71% of the WBV exposure measurements were above the vibration exposure action value of 0.5 m/s^2^ A(8) in the mining sector.

**Table 3:** Summary of whole-body vibration (WBV) exposure metrics [A*w*, A(8) and crest factor] for workers in the mining and transportation sectors.

**Sector (*N*)**	**Parameter**	**Frequency-weighted r.m.s (m/s** ^**2**^ **) acceleration values**		Crest factor value^**^
		A*w*	A(8)	
Mining (*n* = 41)	Mean	0.35	0.63	7.12
	Median	0.22	0.69	7.21
	Standard deviation	0.26	0.20	2.21
	Minimum	0.05	0.08	3.65
	Maximum	1.10	1.42	15.28^*^
Transportation (*n* = 100)	Mean	0.50	0.51	4.93
	Median	0.48	0.50	4.12
	Standard deviation	0.09	0.08	1.88
	Minimum	0.26	0.41	3.16
	Maximum	0.67	0.65	8.26

^*^Only four 9.8% of measurements had a CF of 9 and above in the mining sector.

^**^CF computed as a ratio between frequency-weighted acceleration of Aw_peak/Aw_r.m.s.

**Figure 1:**
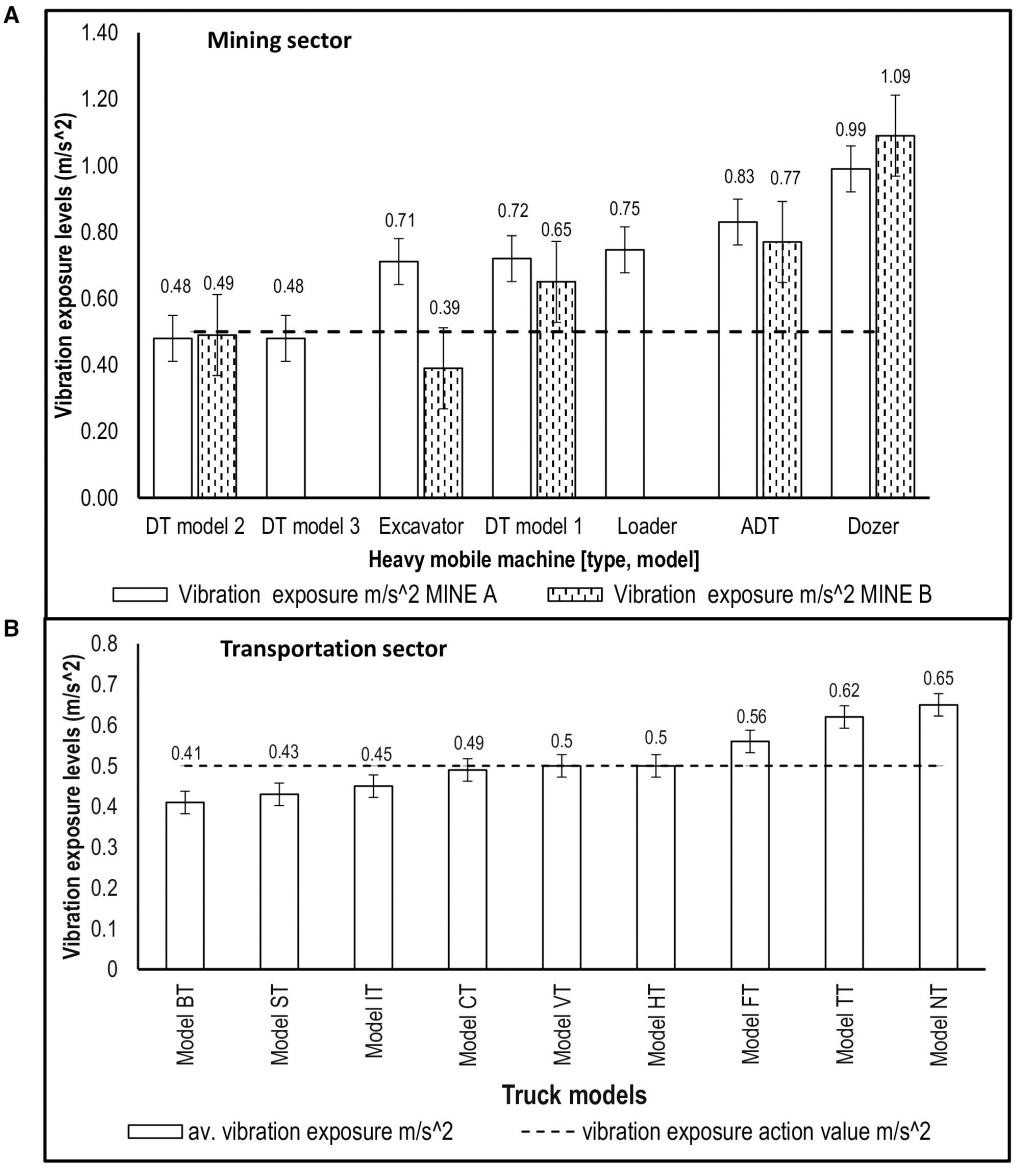
Whole-body vibration exposure [A8, m/s^2^ A(8)] by equipment model in the mining (mines **A** and **B**) and by truck models in the transportation sectors.

Furthermore, at mine site A, the average 8-h WBV exposure level was highest for the dozer operators [0.99 m/s^2^ A(8)], followed by the ADT operators [0.77 m/s^2^ A(8)], and lowest for the excavator operators [0.39 m/s^2^ A(8)]. Similarly, for mine site B, the average 8-h WBV exposure level was highest for the dozer operators [1.09 m/s^2^ A(8)] and lowest for the DT-model-2 and DT-model-3 operators [0.48 m/s^2^ A(8)] (Figure 1). On the other hand, for the transportation sector, the 8-h WBV exposure level was high among model CT truck drivers, i.e., 0.65 m/s^2^,A(8) and lowest among model BT truck drivers, i.e., 0.41 m/s^2^ A(8) (Figure 1). The average 8-h WBV exposure for the five truck models (Model VT, Model HT, Model FT, Model TT, Model NT) of the nine truck models surveyed was 0.5m/s^2^ A(8) and above.

### 3.3 Prevalence of chronic low back pain in relation to characteristics of heavy mobile machine operators and truck drivers

The 12-month prevalence of chronic low back pain was 42.5%. The final modified Poisson regression model revealed that variables such as whole-body vibration, leg symptoms, employment sector-work in the mining sector, years employed, and an increase in BMI were significantly associated with 12-month chronic back pain (Table 4).

**Table 4:** Factors associated with 12-month chronic low back pain among heavy mobile machine operators in mining and truck drivers in the transportation sector in Tanzania (*N* = 632).

**Variable**		**Chronic low back pain**			
	^**^ **Univariable model**		***P*** **-value**	**Multivariable model (*****N*** = 612)^***^		***P*** **-value**
	**Crude RR**	**95% CI**		**Adj. RR**	**95% CI**	
**Whole body vibration exposure [m/s** ^2^ **A(8)]**						
< 0.5	1			1		
≥0.5	1.15	0.84–1.25	0.121	1.22	1.01–1.47^*^	0.039
**Number of resting breaks**						
≤ 2 times	1			1		
3 times or more	0.98	0.80–1.20	0.850	0.92	0.74–1.14	0.441
**Leg pain symptoms**						
no	1			1		
yes	2.46	2.17–2.78^*^	< 0.001	2.36	2.04–2.73^*^	< 0.001
**Backbone and shoulder activities**						
no	1			1		
yes	1.06	0.80–1.40	0.40	1.03	0.79–1.34	0.848
**Age group (years)**						
22–39	1			1		
40–71	1.19	0.9–1.44	0.067	1.07	0.88–1.31	0.482
**Do physical exercises**						
no	1			1		
yes	0.84	0.70–1.01	0.066	0.88	0.73–1.05	0.155
**Employment sector**						
Transportation	1			1		
Mining	1.28	1.01–1.62^*^	0.038	1.36	1.05–1.75^*^	0.012
**Educational level**						
Primary education	1			1		
Secondary education	0.87	0.71- 1.07	0.193	1.01	0.82–1.26	0.139
College/vocational training	0.92	0.66–1.27	0.593	0.95	0.70–1.28	0.327
Body mass index	1.02	1.00–1.04^*^	0.036	1.02	1.01–1.04^*^	0.011
**Years employed (years)**						
0–5	1			1		
6–40	1.33	1.11–1.60^*^	0.044	1.22	1.01–1.47^*^	0.036

^*^RR = Relative Risk from modified Poisson regression with robust variance; 95% Confidence interval, statistically significant at P < 0.05-two sided.

^**^Multivariable “N” reflects complete case observations. Variable specific denominators for descriptive statistics are shown in Table 2.

^***^Complete-case analysis due to missing covariates.

Specifically, workers exposed to WBV ≥0.5 m/s^2^A(8) had a 22% higher relative risk of 12-month chronic low back pain compared to those exposed to < 0.5 m/s^2^ A(8) (adjusted RR 1.22; 95% CI 1.01- 1.47). Reporting leg pain symptoms was associated with more than twofold higher relative risk of 12-month chronic low back pain (adjusted RR 2.36; 95% CI 2.04- 2.73). Compared with working in the transportation sector, working in the mining sector had 36% higher relative risk of 12-month chronic low back pain (adjusted RR 1.36; 95% CI 1.05- 1.75). Longer employment duration of 6–40 years was also associated with a 22% higher relative risk for chronic low back pain compared with 0–5 years (adjusted RR 1.22; 95% CI 1.01- 1.47) (Table 4).

## 4 Discussion

The findings from the present study show that a significant number of heavy mobile machine operators and long-haul truck drivers from the mining and transportation sectors, respectively, in Tanzania were exposed to whole-body vibration levels above the exposure action value [0.5m/s^2^ A(8)]. Furthermore, the prevalence of 12-month chronic LBP was 42.5%, suggesting substantial symptom burden in this workforce. Notably, an increase in BMI, leg pain symptoms, and employment duration were significant factors associated with chronic LBP. This study is among the first in Sub–Saharan Africa and adds to our knowledge of WBV exposure in the mining and transportation sectors, which may help in designing and implementing effective prevention measures and thus mitigate the impact of LBP among workers. However, because the study design was cross-sectional and prevalence of 12- month chronic low back pain was self-reported, causality cannot be inferred.

Prolonged exposure to WBV above 1.15m/s^2^A(8) has been associated with WRMSDs such as chronic LBP (39). In the present study, one-third of all WBV exposure measurements were above the Directive 2002/44/EC—exposure action value (EAV), and close to three-quarters of the measurements in the mining sector were above the EAV, analogous to the studies performed among mobile machine operators in three coal open-pit mines and iron ores in Turkey and India (40, 41). Furthermore, all WBV measurements for dozer operators were the highest and above the EAV, similar to the results reported in the study conducted in two surface coal mines in India, possibly because of the heavy-duty tasks they perform and the rough terrain they operate on coupled with the high frequency of changes in directions, including back and forth movements (13). The average 8-h WBV exposure level varied among the different models across the various models included in the current study. These findings indicate that higher WBV exposure and a substantial prevalence of 12-month chronic LBP co-occur in this workforce; however, given the cross-sectional design and the ISO 2631-1:1997-based exposure assessment, we cannot infer spinal injury mechanisms or degenerative pathology (42). As a result, heavy mobile machine operators and truck drivers are still likely to be at increased health effects from WBV at work despite advancements in technological investments. Nevertheless, these interpretations should be made with caution because this study was cross-sectional and the musculoskeletal outcomes were self-reported. Moreover, ISO 2631-1:1997 RMS-based metrics support exposure characterization and general health guidance but do not quantify cumulative spinal loading or provide direct evidence of spinal injury mechanisms. The current study found lower prevalence of musculoskeletal disorders associated with low back pain than did studies conducted among dump truck operators in coal mines in India, i.e., 42.5% vs. above 80% (13, 43). The reported differences in prevalence might be due to differences in mine characteristics and related tasks; road conditions; other associated factors, such as extended work hours; higher body mass indices; ergonomic factors, such as heavy lifting; driving postures; repetitive work; and psychological factors (10, 44, 45). However, the current study did not cover the details of all these factors.

In the present study, several factors, such as whole-body vibration, leg symptoms, years employed, and an increase in BMI, were significantly associated with self-reported chronic LBP among operators and truck drivers, which is analogous to the findings of other studies (35, 45). While BMI may plausibly influence spinal loading, our result and ISO 2631-1:1997 RMS metrics cannot test degenerative mechanisms (24). Nevertheless, an analysis in an Indian study using machine learning models on multiple documented factors did not find it to be statistically significant (46). Furthermore, in some instances, chronic low back pain is associated with leg symptoms. This may be partly explained by the fact that chronic low back pain may involve neuropathic pain that can radiate from the low back into the legs (resulting in leg symptoms) or may be due to heavy physical tasks such as heavy load lifting, frequent bending and/or frequent working in awkward postures, causing nerve and muscle compression and contributing to the development of chronic low back pain among exposed individuals (47–49). Therefore, the observed association likely reflects shared symptomatology clustering rather than an independent etiologic factor and may be best interpreted as a co-occurring feature of the low back pain syndrome. These findings suggest that prompt management of leg symptoms may be beneficial for workers who are likely to develop work-related chronic low back pain.

In this study, we acknowledge certain limitations. First, the study period, i.e., the measurements of the WBV exposures, did not cover all seasons of the year and may be assumed to be representative of these working conditions. However, we believe that the WBV does not vary significantly with season (17). Second, the frequency-weighted root mean square (RMS) values presented in this study were based on 8 h of daily exposure. In real work situations, mining equipment operators work 12-h shifts. When travel time and meal breaks are factored in, operators are likely to work for approximately 10 h. Thus, the WBV measurement results might underestimate occupational WBV exposures associated with this type of work. To minimize this error, time adjustments were applied in this study. Third, there might be potential sampling bias because heavy machine mobile equipment and trucks were not randomly selected from the mining fleet. However, multiple measurements were collected from each available and well-functioning model and controlled during analyses, which hopefully reduced the potential biases. Fourth, the WBV measurement duration included the complete cycle (loaded and unloaded) without separating specific tasks such as mine materials or mine ore loading and unloading, tipping, road haulage and driving behavior and postures. These factors might have contributed differently to the measured WBV values. Fifth, while ISO 2631-1:1997 is widely used to characterize whole-body vibration (WBV) exposure using frequency-weighted acceleration metrics, it may underestimate exposure severity in occupations where vibration is dominated by repeated shocks and transients. ISO 2631-5:2018 provides a complementary approach specifically intended for WBV exposures containing multiple mechanical shocks, with outputs designed to inform assessment of lumbar spine (vertebral end-plate) compressive loading risk in seated operators (21). Recent field studies applying ISO 2631-5:2018 alongside ISO 2631-1:1997 suggest that risk classification can differ depending on whether exposure is evaluated using RMS or Vibration Dose Value (VDV)-based metrics or shock-focused biomechanical metrics, underscoring the importance of method selection for health-related interpretation (24). However, we believe that the total number of measurements conducted provided adequate vibration information that enabled analysis of WBV in the mining and transportation sectors. Therefore, findings of the current study may be a representation of mining and transportation sectors in the developing countries with similar characteristics.

## 5 Conclusion

This study documented a high proportion of WBV exposure levels above the vibration exposure action value set by the European Directive 2002/44/EC. The study documented the considerable self-reported prevalence of chronic low back pain, suggesting a potential relationship. These findings suggest that a certain percentage of workers are likely to develop vibration-related low back pain and could benefit from preventive interventions at work. Owing to the complexity and interplay of various factors related to WBV exposure and the 12-months chronic low back pain, planning and executing workplace preventive interventions aimed at reducing vibration exposure would be useful. Further studies with WBV repeated measurements are likely to provide more details on the characteristics and variability of vibration exposure together with the clinical assessment of chronic LBP. In addition, future studies could strengthen symptom characterization by incorporating a standardized neuropathic pain screening instrument, for example, the Douleur Neuropathique 4 (DN4) alongside musculoskeletal symptom questions, to better phenotype low back pain with radiating leg symptoms.

## Data Availability

The raw data supporting the conclusions of this article will be made available by the authors, without undue reservation.
